# Overexpression of KIAA0101 Promotes the Progression of Non-small Cell Lung Cancer

**DOI:** 10.7150/jca.45962

**Published:** 2020-09-23

**Authors:** He Cao, Jing Zheng, Yinan Yao, Qi Yang, Runlan Yan, Wenjia Sun, Kexin Ruan, Jianya Zhou, Jianying Zhou

**Affiliations:** 1 Department of Respiratory Disease, Thoracic Disease Center, The First Affiliated Hospital, College of Medicine, Zhejiang University, Hangzhou 310003, China; 2 Department of Respiratory Disease, The First Affiliated Hospital of Jiaxing, Jiaxing University, Jiaxing 314000, China

**Keywords:** KIAA0101, non-small cell lung cancer, diagnosis, prognosis, proliferation

## Abstract

Lung cancer is the leading cause of cancer related death worldwide, with a continue-rising incidence. The proliferating cell nuclear antigen binding protein KIAA0101 is highly expressed in various types of cancer, including non-small cell lung cancer (NSCLC). However, its biological role and underlying mechanisms in NSCLC remains unclear. We downloaded KIAA0101 mRNA and clinical data from The Cancer Genome Atlas (TCGA) and Gene Expression Omnibus (GEO), and verified the KIAA0101 expression by conducting experiments of immunochemistry (IHC), immunofluorescence (IF), quantitative real-time PCR (qRT-PCR) and western-blot. Functional experiments were performed to explore the biological roles *in vitro* and *in vivo*. The results showed that KIAA0101 was overexpressed in NSCLC tissues and cell lines. High KIAA0101 expression was associated with high T stage, nodal invasion, advanced tumor stage, and poor overall survival (*P*<0.01). The receiver operating characteristic (ROC) curves showed that KIAA0101 could distinguish NSCLC from paired normal tissues with statistical significance (AUC=0.969, *P*<0.001). The multivariate analysis revealed that KIAA0101 was an independent prognostic factor for overall survival (HR=1.249, 95% CI: 1.001-1.559, *P*=0.049). Furthermore, KIAA0101 knockdown induced G1 phase cell cycle arrest and inhibited NSCLC cell proliferation and migration. We also found that the depletion of KIAA0101 decreased tumor volume in nude mice. In summary, our findings suggested that KIAA0101 was a reliable diagnostic and prognostic factor in NSCLC, with potential to be a promising treatment target.

## Introduction

Lung cancer ranks as the most common cancer worldwide, with lethal outcomes for the metastasis cases. According to GLOBOCAN 2018, the estimated new lung cancer-related death will reach 1.8 million, accounting for 18.4% of the total cancer-related death 1. In the absence of obvious symptoms in the early phase, the majority of patients with lung cancer are diagnosed in the advanced stage. Conventional treatments, such as pneumonectomy, lobectomy, radiotherapy, and chemotherapy, show limited efficacy and remain unsatisfactory for patients with metastatic non-small cell lung cancer (NSCLC). In the recent decade, the introduction of immunotherapy into clinical practice has transformed the treatment options with tremendous improvements in the prognosis of patients with tumor disease. However, the death rate of metastasis lung cancer still remains at a relatively high level. Therefore, reliable biomarkers and promising therapeutic targets are urgently needed to inspire novel treatments for lung cancer.

KIAA0101, also known as proliferating cell nuclear antigen (PCNA)-associated factor (PAF15), is a conserved PCNA-binding protein that plays an essential role in the regulation of various biological processes 2-4. KIAA0101 protein competes with the binding of DNA polymerase to regulate DNA replication and damage repair. Recently, KIAA0101 has been reported to play a pro-cancer and anti-cancer role in cancer development. For example, KIAA0101 knockdown significantly induces G1/S phase arrest and inhibits cell growth in breast cancer cells 5. Furthermore, miR-197-5p targets KIAA0101 to induce cellular senescence in fibrosarcoma 6. In contrast, KIAA0101 level is decreased in hepatocellular carcinoma (HCC) tissues, and KIAA0101 cDNA transfection can inhibit cell growth *in vitro* 7. In summary, the functions of KIAA0101 differs in different types of tumors. A recent study revealed that KIAA0101 expression is upregulated in NSCLC tissue, and patients with high KIAA0101 expression are associated with early postoperative recurrence 8. However, the biological functional analysis of KIAA0101 in lung cancer cells has never been performed.

Therefore, we aim to investigate whether KIAA0101 expression is associated with clinicopathological characteristics and survival time of patients with NSCLC. Besides, the biological role of KIAA0101 was also explored *in vitro* and *in vivo*.

## Materials and methods

### NSCLC tissue samples

A total of 11 pairs of NSCLC tissues and adjacent normal tissues were collected from Jan. 2018 to Mar. 2018. These patients received surgical lobectomy or pneumonectomy at the First Affiliated Hospital of Zhejiang Medical College, and cases pretreated with radiotherapy and/or chemotherapy were excluded. The study was approved by the Human Research Ethics Committee of the First Affiliated Hospital of Zhejiang Medical College. Written informed consent was provided.

### Cell lines

We purchased HEK293T and NSCLC cell lines (A549, H1299, PC-9, and H226) from American Type Culture Collection (ATCC, Manassas, VA), and obtained MRC-5, a human non-malignant cell line, from the Cell Bank of the Chinese Academy of Medical Sciences (Shanghai, China). H1299 and H226 cells were cultured in RPMI-1640 (Hyclone, Logan, Utah, USA) with 10% fetal bovine serum (FBS, Gibco, Grand Island, NY, USA), and other cell lines were cultured in Dulbecco's Modified Eagle Medium (DMEM, Hyclone, Logan, Utah, USA) containing 10% FBS and 1% penicillin-streptomycin solution. All cells were maintained in an incubator at 37 °C with 5% CO_2_.

### RNA isolation and quantitative real-time PCR (qRT-PCR)

Total RNA was extracted using TRIzol reagent (Invitrogen, Carlsbad, CA), following the manufacturer's protocols. Then we carried out complementary DNA (cDNA) synthesis with a PrimeScript™ RT reagent Kit (Takara, Dalian, China). Quantitative real-time PCR (qRT-PCR) analysis was performed in a CFX96 real-time PCR Detection System (Bio-Rad, California, USA) using SYBR^®^ Premix Ex Taq™ II master mix (Takara, Dalian, China). Each test was repeated for three times, and GAPDH was used as an internal control. Relative mRNA expression was calculated using the 2^-ΔΔCt^ method. All sequences of the primers are listed in Table 1.

### Western Blot

Cells were washed by pre-cold PBS twice, followed by lysis with cold RIPA (Beyotime, Shanghai, China) containing phosphatase inhibitors and protease inhibitors. Protein concentration was calculated using the BCA method. Total proteins were electrophoresed by 12% SDS-PAGE gel and were transferred onto polyvinylidene fluoride (PVDF, Millipore, Bedford, MA, USA) membranes. Next, the membranes were blocked in 5% skimmed milk powder and were incubated overnight at 4 °C with the primary antibody against KIAA0101 (1:1000, Abnova), cyclin D1 (1:1000, Cell Signaling Technology), cyclin B1 (1:1000, Cell Signaling Technology), CDK2 (1:1000, Cell Signaling Technology), CDK6 (1:1000, Proteintech) and α-tubulin (1:5000, Proteintech). After that, membranes were incubated with an HRP-conjugated second antibody (1:20000, Beyotime, Shanghai, China) for 1h at room temperature. After a wash with 0.1% TBST for three times, target proteins were visualized using an enhanced chemiluminescence system (Bio-Rad, California, USA).

### Plasmid construction and cell transfection

The pLKO.1 plasmid vector (Sigma Aldrich, St Louis, MO, USA) was used to knock down KIAA0101 expression. The targeted sequence and scramble shRNA sequence were synthesized (Tsingke, Hangzhou, China) and then were subcloned in pLKO.1 vector. The detailed shRNA sequences are listed in Table 1. A total of 5×10^5^ cells were seeded into six-well plate overnight, and were transfected with 2μg plasmid using Lipofectamin® 3000 reagents (Invitrogen, Carlsbad, CA) according to manufacturer's instructions. At 72h after transfection, we performed western blot to determine the inhibition efficiency.

### Lentivirus package and stable cell culture

For experiments *in vivo*, we also constructed stable-transfected cells. Lentivirus was packaged in HEK293T with 10μg pLKO.1, 10μg pspax2, and 10μg pMD2G plasmids. The cell suspension was centrifuged at 3000rpm to collect virus suspension. Then we mixed PEG8000 with the suspension to purify the virus. To construct a stable-transfected cell line, we infected cells with 200μl lentivirus and selected with 2μg/mL puromycin (GeneChem, Shanghai, China).

### Immunohistochemistry

Four to five micrometer thick formalin-fixed paraffin-embedded (FFPE) sections were deparaffinized and rehydrated in graded solution. After antigen retrieval, sections were incubated overnight at 4°C with the KIAA0101 primary antibody (1:200, Abnova, Taiwan, China). After a wash with PBS for three times, goat anti-mouse IgG was incubated for 1h at 37°C. Then, DAB (OriGene, Beijing, China) staining was performed to visualize peroxidase activity. Images were observed with a light microscope (Olympus Corporation, Tokyo, Japan, magnification×400).

### Immunofluorescence preparations

Seeded cells were fixed with 4% paraformaldehyde for 30min. After washing for three times with PBS, 0.25% Triton X-100 was added for permeabilizing membrane. Then cells were blocked with 3% BSA for 1h, and were incubated with the KIAA0101 antibody (1:200, Abnova, Taiwan, China) at 4 °C overnight. On the next day, cells were incubated with Alexa Fluor 594 goat anti-mouse antibody (KeyGen, Nanjing, China). DAPI dilution (Invitrogen, NY, USA) was incubated to visualize the nuclei. Images were observed with a light microscope (Olympus Corporation, Tokyo, Japan, magnification×400).

### CCK-8 and cell colony formation assay

The CCK-8 assay was used to assess cell proliferation ability (Dojindo, Kumamoto, Japan). A density of 1000 cells were seeded in 96-well cell plate after 48h transfection. In order to test the proliferation ability, 10μl CCK-8 reagent was added to each well and cells were cultured at 37°C incubator for 1 hour. The optical density (OD) values of 450nm were measured at 24h, 48h, 72h, and 96h, respectively. For cell colony formation, single-cell suspensions were seeded into six-well plates (1000 cells/well). After 10-14 days of cell culture, cells were fixed with 4% paraformaldehyde and stained with crystal violet solution.

### Transwell migration and wound healing assay

For the transwell migration assay, approximately 1×10^4^ cells were seeded into the upper chambers with serum-free DMEM. The lower chambers were filled with 700μl DMEM containing 10% FBS. After incubation for 24h, we removed the cells in the upper side and fixed the cells in the bottom chambers, then stained them with crystal violet. Stained migration cells selected from 3 random fields were pictured and counted. A wound-healing assay was also used to test the migration ability. Cells were scratched using 10μl sterile tips when they reached 90% confluence. After a wash with PBS twice, the culture medium was replaced with serum-free DMEM. Photos were recorded every 12h with a microscope (Olympus Corporation, Tokyo, Japan, migration×100).

### Cell cycle assay

Cells were harvested after a transfection with shRNA for 48h and were fixed with pre-cooled ethanol for over 4h. After being washed with PBS, cells were stained with propidium iodide/RNase (Yeasen, Shanghai, China) for 15min in dark. Then cell cycle analyses were performed by flow cytometry (FACS Verse, BD Biosciences, MA, USA).

### Tumor in vivo

Male nude mice (4-6 weeks old) were purchased from Shanghai SLAC Laboratory Animal Co. Ltd. The mice were randomly divided into two groups, with three mice in each group. After a week adjustment to local conditions, mice were subcutaneously implanted with 1×10^6^ PC-9 cells. The sizes of xenograft tumors were recorded every 2-3 days. The tumor volume was calculated as 0.5×length×width^2^. The Ethics Committee approved all animal studies.

### Bioinformatic analysis of public databases

We downloaded the level 3 data from The Cancer Genome Atlas (TCGA, https://portal.gdc.cancer.gov/). GSE19804 and GSE18842 were also downloaded from Gene Expression Omnibus (GEO) (https://www.ncbi.nlm.nih.gov/geo/). Data in count form was normalized into log2 form for analysis.

### Gene set enrichment analysis (GSEA)

To identify relevant biological pathways, we performed GSEA to determine a significant and concordant difference that existed between two types of physiological states. A gene set was considered to enrich when the standard *P*-value was <0.05 and the false discovery rate (FDR) was <0.25.

### Statistical analysis

Statistical analyses were performed with SPSS software 22.0 and GraphPad Prism 7.0 (La Jolla, CA, USA). The differences between categorical variables were compared with a chi-square test. The receiver operating characteristic (ROC) curves and area under the curve (AUC) were used to calculate the diagnostic value in patients with NSCLC. Survival curves were performed using the Kaplan-Meier method with the log-rank test. Next, univariate and multivariate analyses were performed to identify the risk factors. A *P*-value less than 0.05 was defined as statistically significant for all analyses.

## Results

### KIAA0101 was significantly overexpressed and associated with clinical parameters in NSCLC

**Figure 1:**
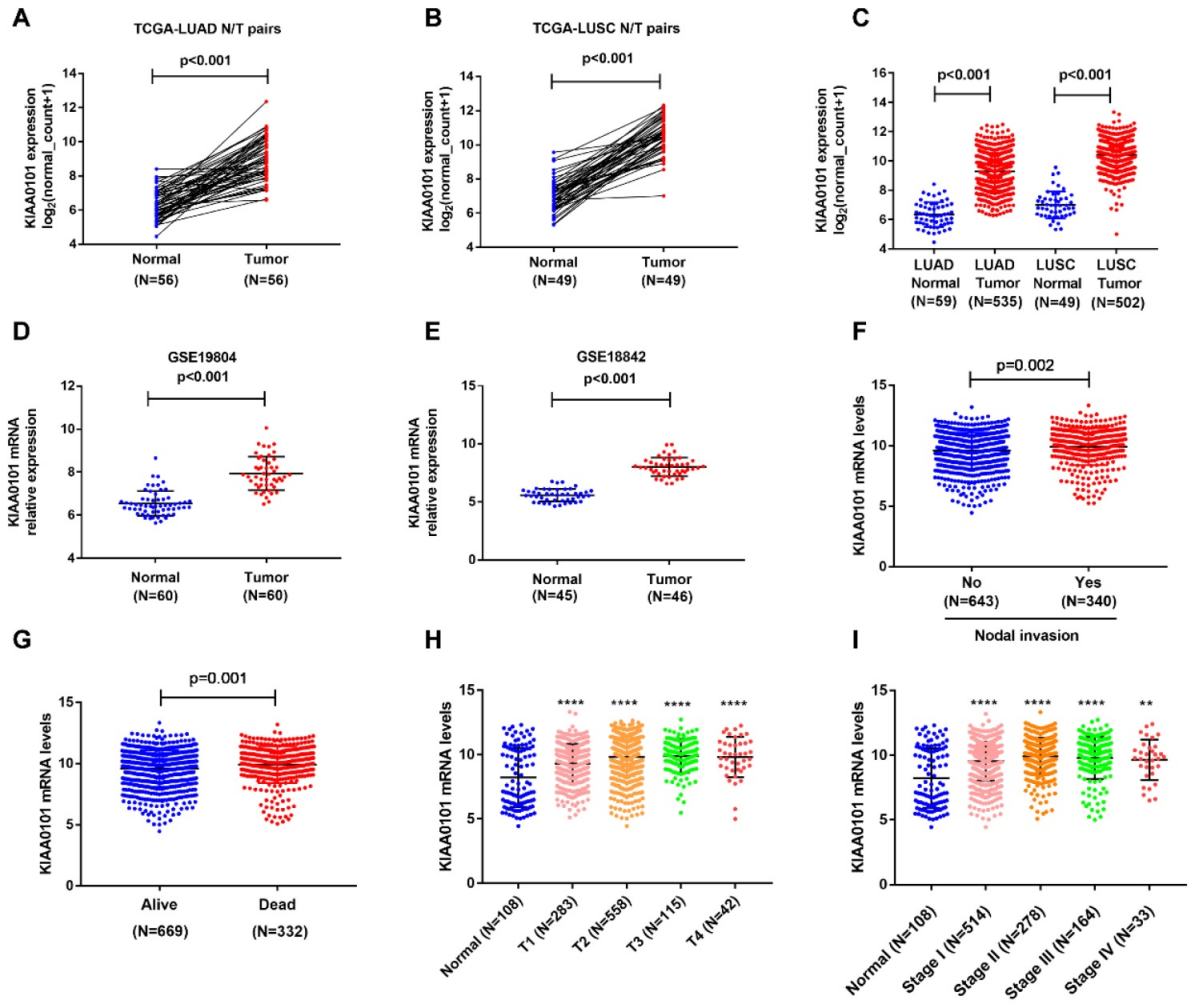
KIAA0101 expression was significantly overexpressed and related to various clinical parameters in NSCLC patients. KIAA0101 expression was upregulated in 56 LUAD (A) and 49 LUSC (B) tissues than paired normal tissues. KIAA0101 mRNA overexpressed in NSCLC tissues in (C) TCGA, (D) Lu lung dataset, (E) Ramos lung dataset. High KIAA0101 expression positively related to various clinical parameters: (F) nodal invasion, (G) poor outcome, (H) advanced T stage, and (I) tumor stage. **,* P*<0.01; ****, *P*<0.0001.

To find out the expression of KIAA0101 in NSCLC, we downloaded mRNA expression data from TCGA, which included 1037 lung tumor tissues and 108 adjacent normal lung tissues. As shown in Figure 1A-C, KIAA0101 was significantly upregulated in 502 LUSC and 535 LUAD tumor tissues compared with that in normal tissues (*P*<0.001). Analysis results from GSE19804 and GSE18842 datasets confirmed the overexpression of KIAA0101 in lung cancer tissues (all *P*<0.001, Figure 1D and E). Next, we assessed the correlation between KIAA0101 expression and different clinicopathological parameters. KIAA0101 upregulation was positively correlated with advanced T stage, nodal invasion, tumor stage, and poor outcome (Figure 1F-I; Table 2). However, no difference was observed between the patients of aged ≥60 and aged <60 (Table 2).

### The prognostic value of high KIAA0101 expression in NSCLC

**Figure 2:**
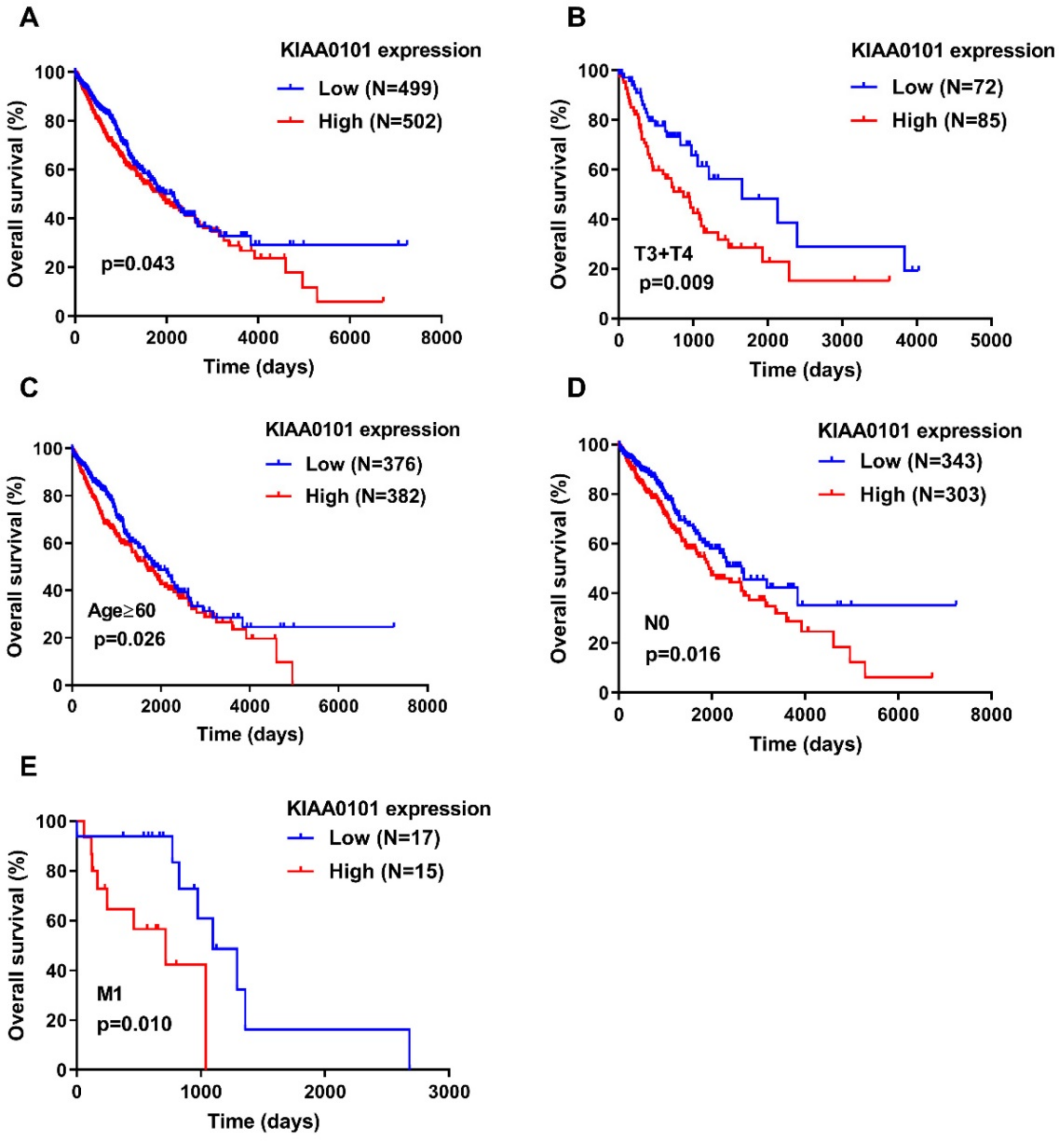
High KIAA0101 expression was associated with a significantly inferior overall survival in NSCLC patients. (A) High KIAA0101 expression group exhibited a significantly inferior overall survival compared with those in the low expression group. Survival analyses were conducted in the subgroups of patients with NSCLC: (B) T3+T4 stage, (C) aged≥60, (D) without nodal invasion, and (E) metastasis.

A Kaplan-Meier survival curves was generated with a log-rank test to explore the association between KIAA0101 expression and overall survival in patients with NSCLC. Figure 2A showed that patients with high KIAA0101 expression exhibited a significantly inferior overall survival compared with those in the low expression group (*P*=0.043). Figure 2B-E indicated that KIAA0101 expression was a powerful predictive tool for prognosis in patients with metastatic NSCLC (*P*=0.010), aged≥60 (*P*=0.026), T3+T4 stage (*P*=0.009), and without nodal invasion NSCLC (*P*=0.016). In addition, Cox regression analysis confirmed that KIAA0101 expression was an independent risk factor for overall survival (HR=1.249, 95% CI: 1.001-1.559, *P=*0.049; Table 3).

### The diagnostic value of high KIAA0101 expression in NSCLC

**Figure 3:**
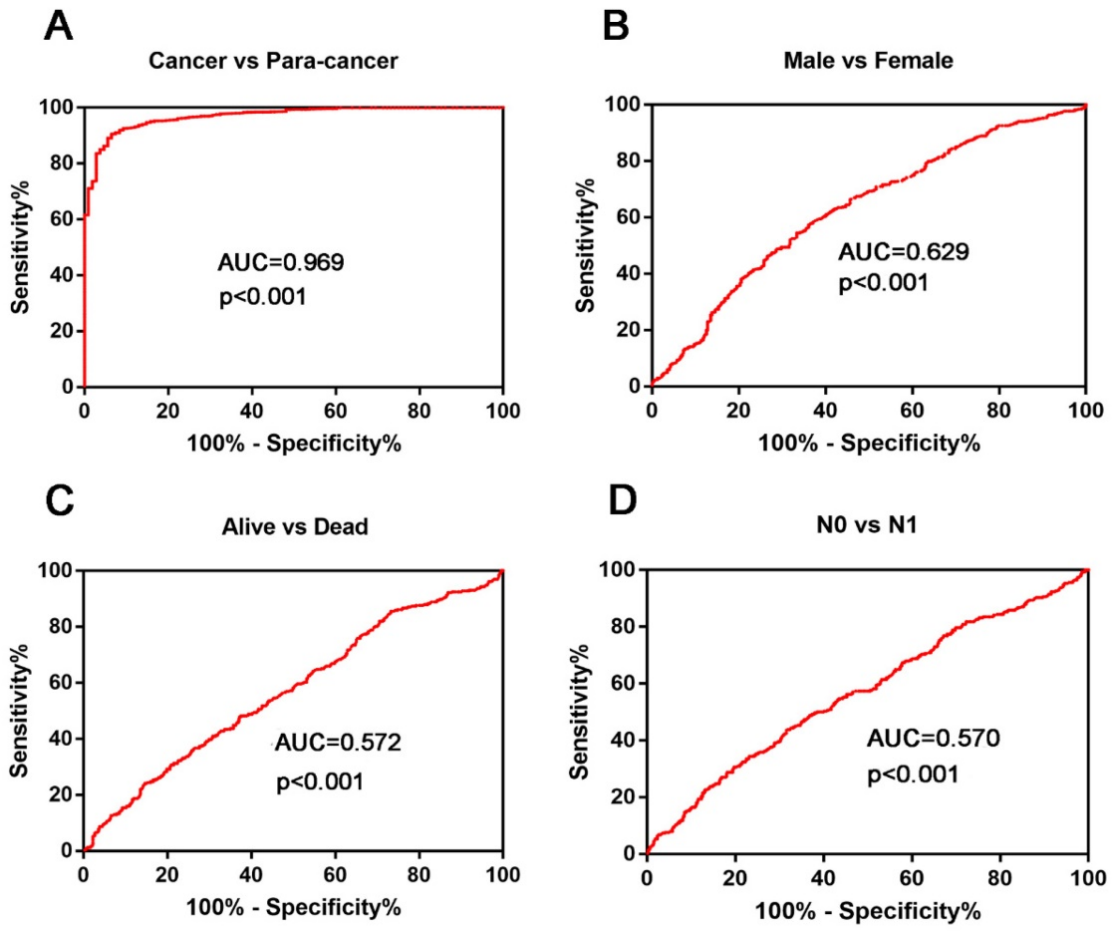
KIAA0101 expression played a potential diagnostic role in NSCLC. (A) KIAA0101 sufficiently distinguish NSCLC from paired normal tissues. ROC subgroup analyses showed that KIAA0101 expression might be a potential prognostic indicator with the following characteristic: (B) Male vs. Female; (C) Alive vs. Dead; (D) N0 VS N1.

ROC curves were used to investigate the diagnostic role of KIAA0101. Results showed that KIAA0101 could sufficiently distinguish NSCLC from paired normal tissues (Figure 3A, AUC=0.969, *P*<0.001). We performed ROC analysis in other subgroups and found that high KIAA0101 expression may be a potential prognostic indicator for patients with following characteristic: Male vs. Female (Figure 3B, AUC=0.629, *P*<0.001); Alive vs. Dead (Figure 3C, AUC=0.572,* P*<0.001); N0 vs. N1 (Figure 3D, AUC=0.570, *P*<0.001).

### KIAA0101 overexpression was verified in NSCLC cell lines and tissues

**Figure 4:**
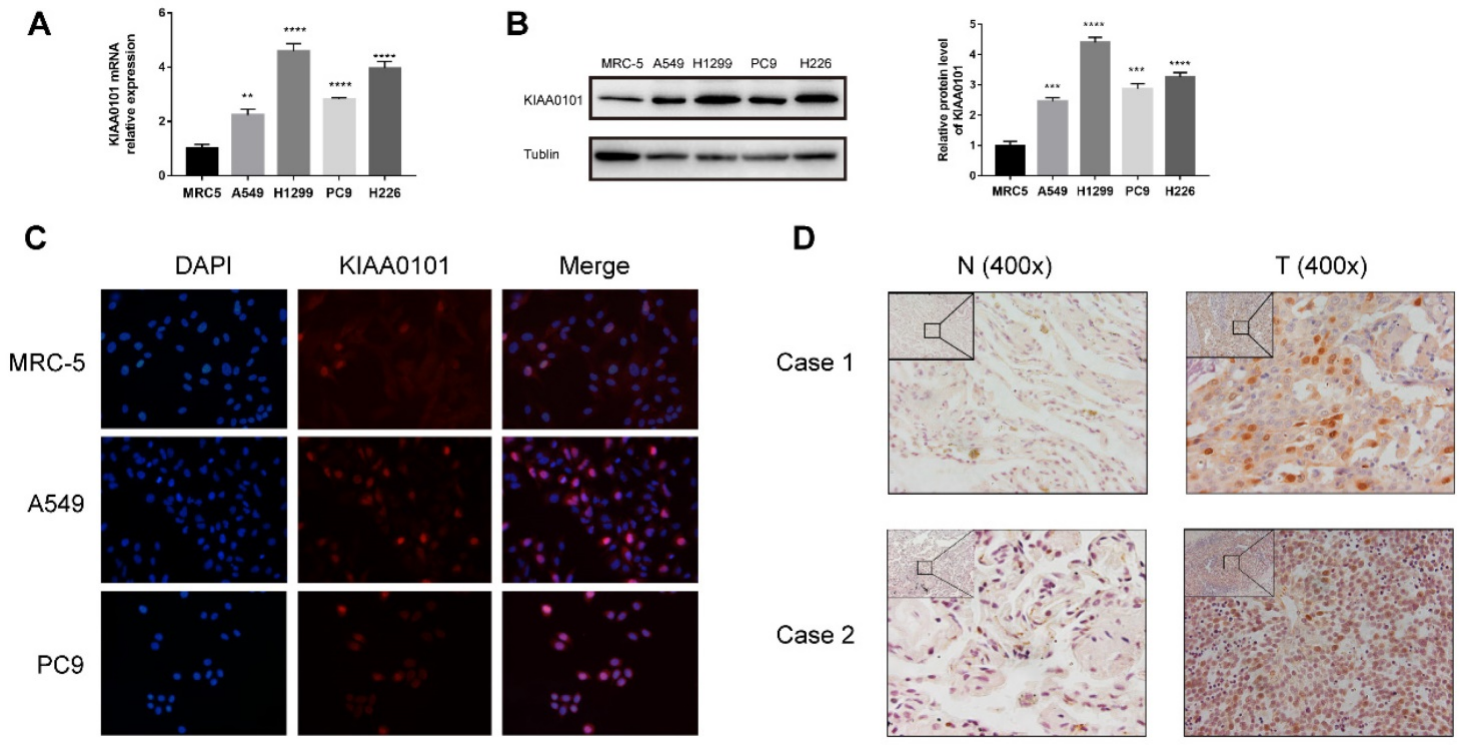
KIAA0101 overexpression was verified in NSCLC cell lines and tissues. (A) qRT-PCR and (B) western-blot results of KIAA0101. (C) Immunofluorescence of KIAA0101 in NSCLC cell lines (magnification×400). (D) Immunochemistry staining of KIAA0101 in NSCLC tissues and adjacent normal tissues (magnification ×100 and×400). **,* P*<0.01;***, *P*<0.001; ****, *P*<0.0001.

To verify the KIAA0101 expression, qRT-PCR and western blot were performed. The mRNA level of KIAA0101 was significantly up-regulated in several lung adenocarcinoma and lung squamous carcinoma cell lines than in the MRC-5 cells (Figure 4A). Western blotting showed similar results in the protein level (Figure 4B). Besides nuclei location in immunofluorescence, we also observed a similar protein expression profile consistent with the western-blot method (Figure 4C). The expression of KIAA0101 was also detected in 11 pairs of NSCLC tissues, KIAA0101 protein mainly located near the nucleus and was overexpressed in eight out of 11 cancer tissues (Figure 4D).

### The knockdown of KIAA0101 expression inhibited NSCLC proliferation *in vitro* and *in vivo*

**Figure 5:**
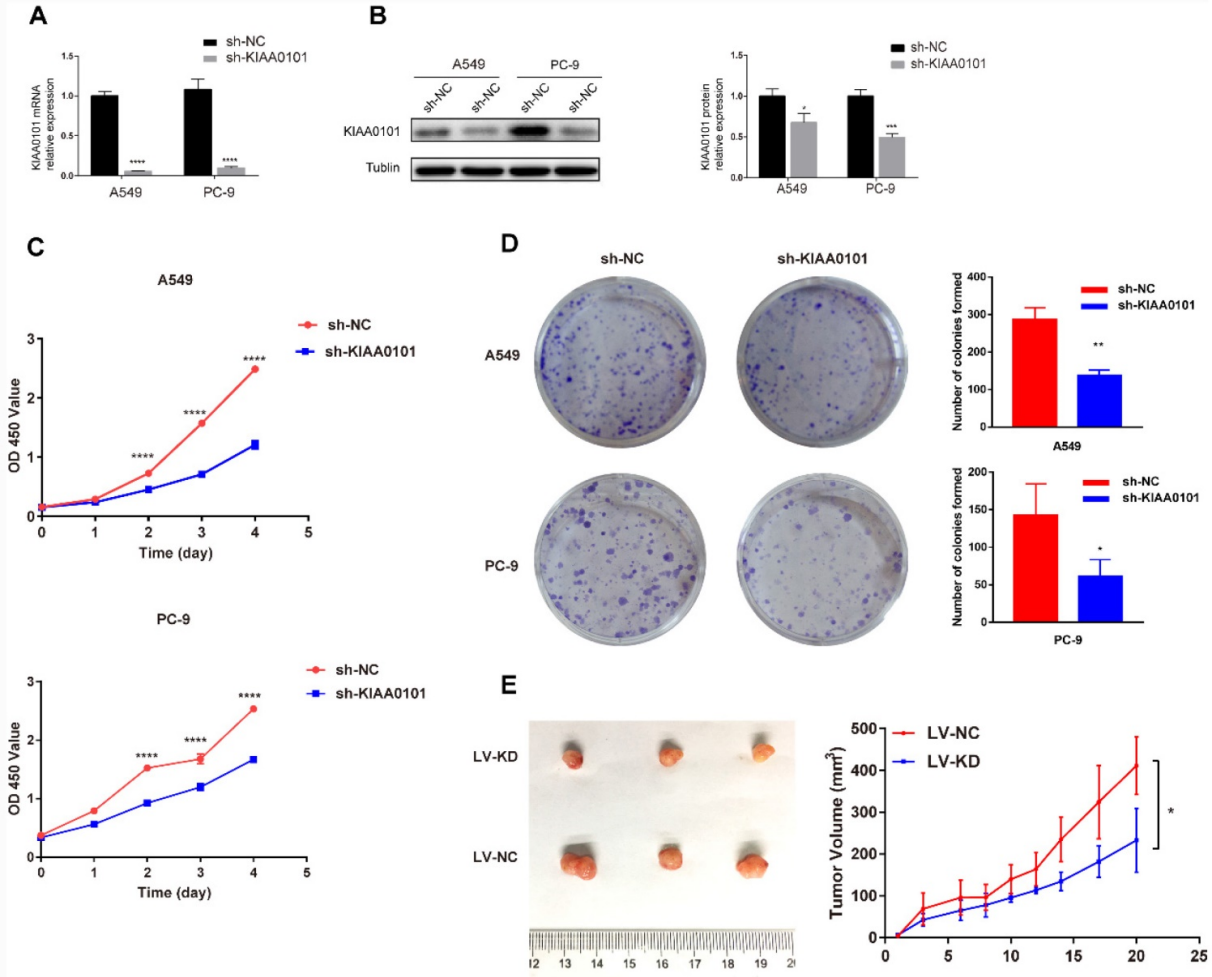
The knockdown of KIAA0101 inhibited NSCLC proliferation *in vitro* and *in vivo*. After transfection for 48h-72h, (A) mRNA and (B) protein levels of KIAA0101 significantly reduced. (C-D) The proliferation capacity of A549 and PC-9 cells decreased according to CCK-8 and colony formation assay. (E) cell growth in the xenograft model. *, *P*<0.05; **,* P*<0.01;***, *P*<0.001; ****, *P*<0.0001.

The shRNA oligos were utilized to silence KIAA0101 expression. After transfection for 48h, mRNA levels of KIAA0101 were significantly decreased compared with the negative control group in A549 and PC-9 cells (*P*<0.001, Figure 5A). Western blot assays showed significant depletions in KIAA0101 protein at 72h (Figure 5B). In the results of CCK-8 assays (Figure 5C), optical density (OD) values in the sh-KIAA0101 group decreased significantly at 24h, 48h, 72h, and 96h (*P*<0.001), respectively. For colony formation assay (Figure 5D), negative control group visualized more colonies after 10-14 days' culture in A549 (285.7±18.8 vs. 137.0±8.7; *P*=0.002) and PC-9 cells (142.3±24.3 vs. 61.3±12.9; *P*=0.042). In the xenograft model, we observed that the sh-KIAA0101 group showed remarkable decrease in tumor volume compared to the control group (Figure 5E).

### The knockdown of KIAA0101 expression inhibited NSCLC migration *in vitro*

**Figure 6:**
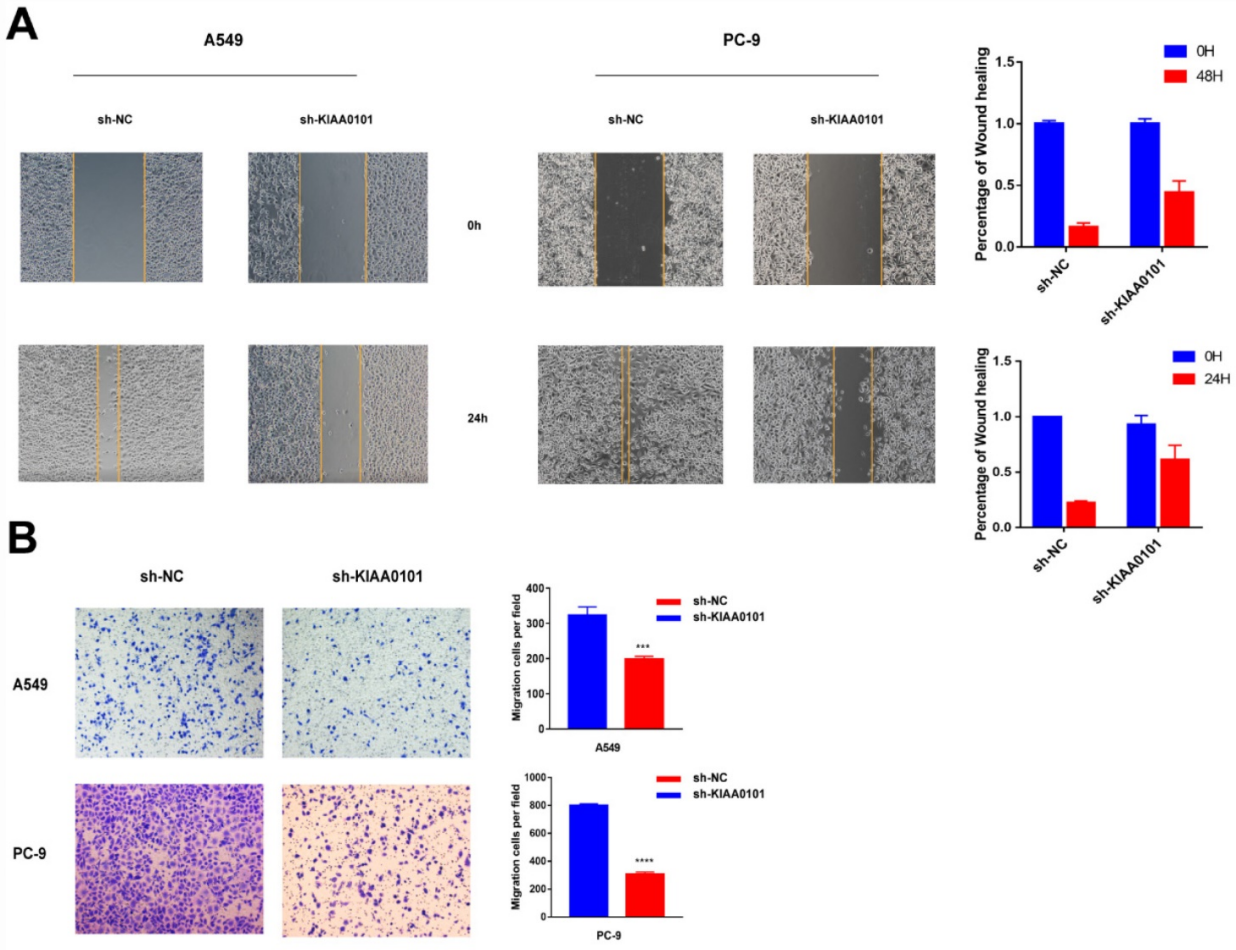
The knockdown of KIAA0101 expression inhibited NSCLC migration *in vitro*. Representative images of woung-healing using (A) A549 and (B) PC-9 (magnification ×100). Representative images of transwell using (C) A549 and (D) PC-9. (magnification×400). ***, *P*<0.001; ****, *P*<0.0001.

To evaluate the effect of KIAA0101 on the ability of migration, wound-healing and transwell assay were used. As shown in Figure 6A, the wound healing rate in the negative control group was 77.8±2.0% in A549 cells and 83.9±5.8% in PC-9 cells after 24h serum-free DMEM incubation. However, the sh-KIAA0101 group only showed a healing rate of 31.5±4.8% (*P*<0.001) and 55.6±10.6% (*P*=0.02). In transwell assay (Figure 6B), migration ability was significantly suppressed after KIAA0101 depletion. The number of migrated A549 and PC-9 cells was 324.3±13.0 and 800.0±7.6, respectively, and the number of migrated cells sharply decreased to 197.3±5.2 and 305.7±9.3 in sh-KIAA0101 group.

### KIAA0101 was involved in the TP53 pathway and cell cycle

**Figure 7:**
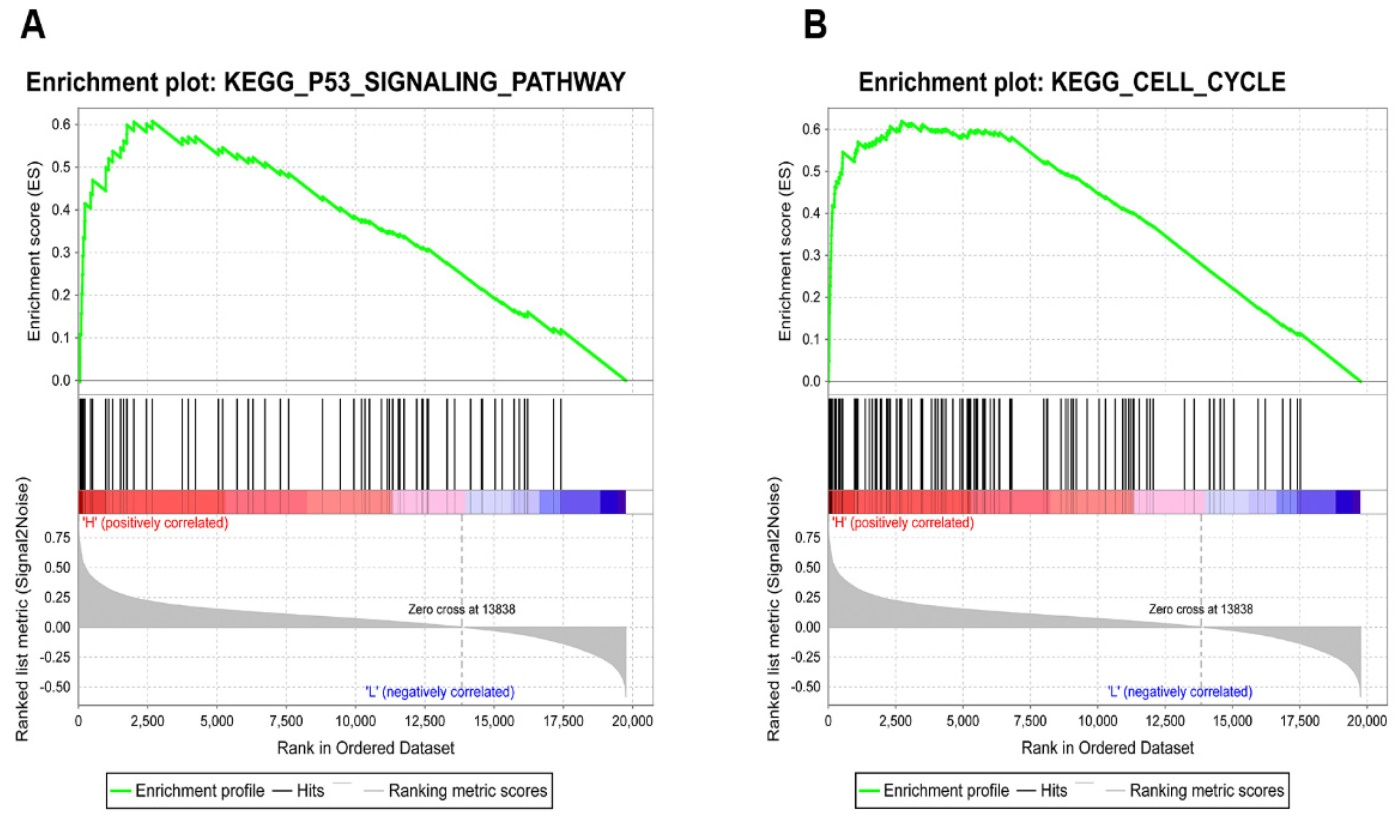
Identification of KIAA0101-related pathways with GSEA. High KIAA0101-related genes significantly enriched in (A) TP53 signaling pathway and (B) cell cycle.

**Figure 8:**
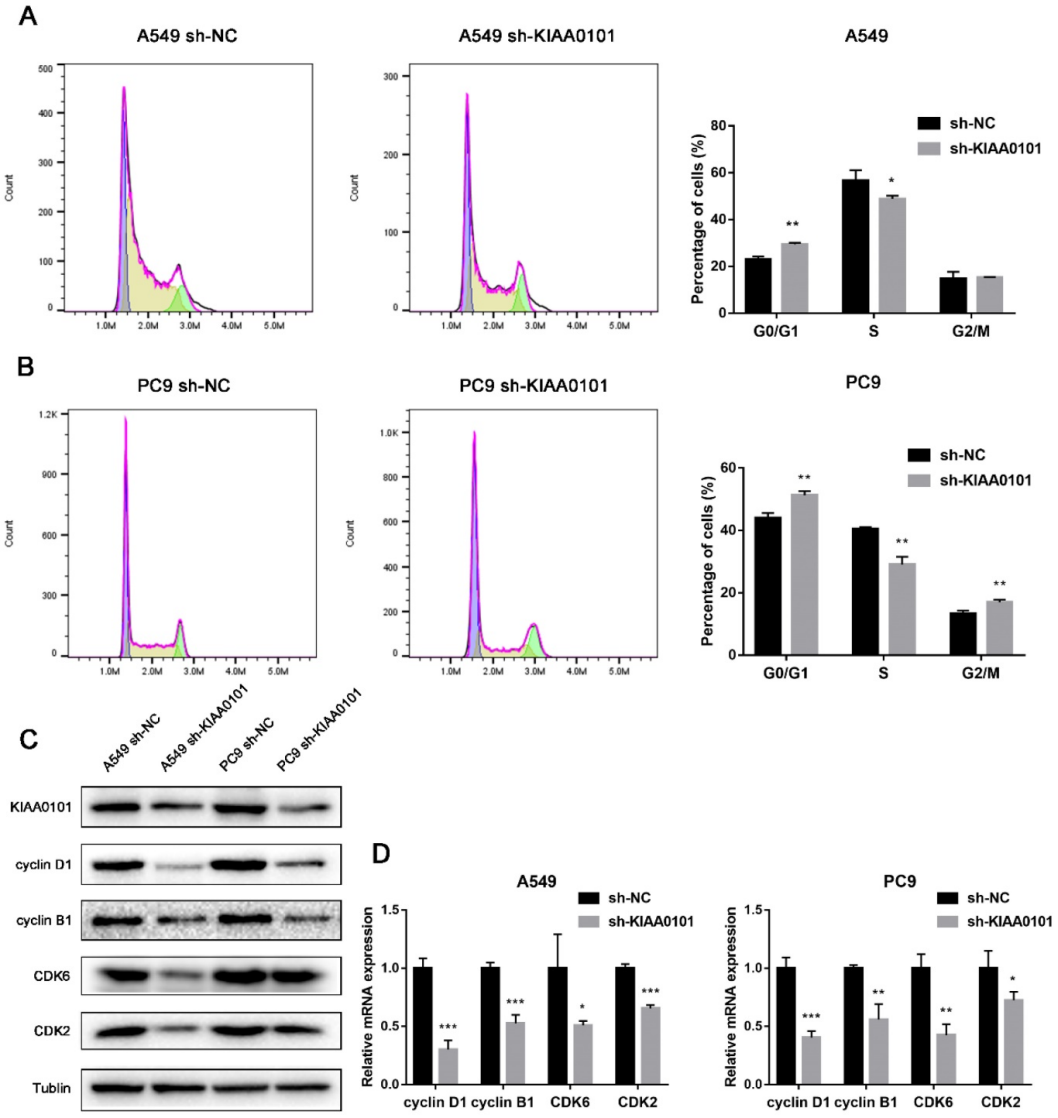
KIAA0101 knockdown induced G1 phase cell cycle arrest. Flow cytomatric analysis of cell cycle distribution of A549 (A) and PC-9 (B) cells. Western blot and qRT-PCR showed decreased expression of cyclin D1, cyclin B1, CDK2, and CDK6 in protein (C) and mRNA level (D). *, *P*<0.05; **,* P*<0.01.

To identify how KIAA0101 influences tumor development, we performed GSEA with TCGA data and used c2.cp.kegg.v6.2.symbols.gmt as the reference gene. Results showed high KIAA0101-related genes were significantly enriched in TP53 signaling pathway (NES=1.95, *P*<0.001, FDR=0.078; Figure 7A) and cell cycle (NES=1.94, *P*<0.001, FDR=0.047; Figure 7B). The influence of KIAA0101 on cell cycle was also investigated by flow cytometry. As shown in Figure 8A-B, KIAA0101 knockdown significantly increased the number of cells in G1 phase compare to the control group (*P*<0.05). At the molecular level, the expression of cyclin D1, cyclin B1, CDK2, CDK6 were decreased in sh-KIAA0101 treated cells (Figure 8C-D).

## Discussion

NSCLC is a common malignancy with high incidence, as the leading cause of cancer-related mortality. Due to the unobvious early symptoms, over 50% of the patients with NSCLC are diagnosed in an advanced stage at the first visit. Traditional surgery and chemotherapies remain unsatisfactory for those patients. Immunotherapy has continued to establish indications expanding across several cancers in recent years and preliminary clinical data suggest that the cytotoxic T-lymphocyte-associated antigen 4 (CTLA-4) and programmed death 1 (PD-1) inhibitors are promising approaches for tumor managements 9. However, the 5-year survival in patients with advanced lung cancer still remains less than 20%. Therefore, novel potential biomarkers and personalized therapeutic targets are urgently needed.

In this study, we found KIAA0101 was overexpressed in NSCLC tissues compare to corresponding normal tissues, and it was useful for diagnosis and prognosis prediction in NSCLC. Besides, the decreased KIAA0101 inhibited cell proliferation and migration abilities, and induced G1 cell cycle arrest in NSCLC cells *in vitro* and *in vivo*.

KIAA0101, also named p15^PAF^ (PCNA-associated factor) and OEATC-1 (overexpressed in anaplastic thyroid carcinoma-1), was first discovered as a conserved protein that interacted with PCNA 2, 10. By competing with PCNA binding, KIAA0101 involves in the regulation processes of DNA replication, DNA repair, and cell cycle 4, 11. Recent researches focus on its oncogenic role and indicate that KIAA0101 level is markedly increased in various types of cancer, including breast 5, adrenal 12, ovarian 13, and gastric cancer 14. In this study, we observed an increased expression of KIAA0101 in NSCLC tissues than in the paired normal tissues as well as in NSCLC cells. TCGA and GEO analysis also confirmed these results. In a previous study conducted by Tatsuya et al. 8, they analyzed the KIAA0101 mRNA expression in 12 NSCLC cases and found that KIAA0101 was overexpressed in eight out of 12 cases, which was consistent with our report.

We discovered that high KIAA0101 expression was positively correlated with nodal invasion, advanced tumor stage, and inferior overall survival, and it was considered as a powerful prognostic indicator for NSCLC. Further analysis showed that KIAA0101 expression and nodal invasion were independent risk factors. Our results were supported by several previous studies with similar results. Abdelgawad et al. revealed that KIAA0101 mRNA in the peripheral blood was associated with distant metastasis and advanced stage in patients with hepatocellular carcinoma. Moreover, KIAA0101 mRNA exhibited better sensitivity and specificity than those of alpha-fetoprotein (AFP) and carcinoembryonic antigen (CEA) 15. In the tumor setting of human gastric cancer, Zhu et al. reported that increased KIAA0101 was a risk marker for recurrence 14. As for the possible mechanism behind the pro-tumor effect, KIAA0101 was reported to be involved in tumor invasion and recurrence by promoting epithelial-to-mesenchymal transition (EMT) by Zhang et al. and Jin et al. 13,16. Based on the prior literature, we speculate that KIAA0101 might partly involve in EMT in NSCLC. Notably, this hypothesis is supported by the fact that after reducing KIAA0101 expression, we observed a decreased migration ability in cells.

The proliferation ability was significantly reduced in A549 and PC-9 cells after knockdown of KIAA0101. Moreover, in the xenograft model, the tumor volume significantly decreased compared to the control group. To further reveal the potential mechanism, we performed GSEA and found that KIAA0101 related genes mostly affected TP53 signaling pathway and cell cycle. As a critical tumor suppressor, wild type TP53 protects cells from oncogenesis by maintaining the genome stabilities 17. TP53 protein activates a panel of genes to repair genome damage when cells are under stress. Also, some studies report that TP53 can interact with some G1 or G2/M checkpoint proteins to induce cell cycle arrest 18. In our study, flow cytometric analyses confirmed that KIAA0101 was involved in NSCLC cell cycle progression, and the depletion of KIAA0101 induced cells cycle arrest in G1 phase. Moreover, we observed a significant decrease in cyclin D1, cyclin B1, CDK2, and CDK6. These proteins tightly control the cell cycle progression, with critical roles in DNA replication and mitosis. On the other hand, they are also important downstream factors in the TP53 pathway 19. Lv et al. reported the network of KIAA0101, TP53, SP1 functioned in breast cancer, and the depletion of KIAA0101 suppressed cell cycle progression. In luciferase reporter assay and ChIP assay, they found KIAA0101 disrupted the interaction between TP53 and SP1 5. Based on these evidence, we assume that KIAA0101 could influence cell proliferation by TP53 network and cell cycle.

To the best of our knowledge, the present study first reported the oncogenic role of KIAA0101 by integrating bioinformatics with functional cell experiments. However, there are some shortcomings in our study. The mechanism of KIAA0101 overexpression remains unclear, and the molecular mechanism to promote cancer progression has not been fully elucidated. More works are required to be done to clarify these issues.

In conclusion, KIAA0101 is overexpressed in NSCLC with important diagnostic and prognostic roles. Furthermore, KIAA0101 promotes cancer progression by enhancing cell migration and proliferation, which provides a new clue to develop more efficient therapies in the future.

## Figures and Tables

**Table 1:** Sequences of primers and shRNA used in this study.

Primers used for quantitative real-time PCR		
Gene	Forward Primer	Reverse Primer
KIAA0101	CTCTGCCACTAATTCGACATCA	TTCAGAATCTTTAGGGGACAAC
Cyclin D1	GCTGCGAAGTGGAAACCATC	CCTCCTTCTGCACACATTTGAA
Cyclin B1	AATAAGGCGAAGATCAACATGGC	TTTGTTACCAATGTCCCCAAGAG
CDK2	CCAGGAGTTACTTCTATGCCTGA	TTCATCCAGGGGAGGTACAAC
CDK6	GCTGACCAGCAGTACGAATG	GCACACATCAAACAACCTGACC
GADPH	TGACTTCAACAGCGACACCCA	CACCCTGTTGCTGTAGCCAAA
shRNA oligos		
KIAA0101	CCGGGCAACCTGATCACACAAATGACTCGAGTCATTTGTGTGATCAGGTTGCTTTTTTG	
Control	TTCTCCGAACGTGTCACGTCTCGAGACGTGACACGTTCGGAGAATTTTTT	

**Table 2:** Comparison of the association between KIAA0101 expression and clinicopathological parameters in patients with NSCLC.

Characteristics	KIAA0101 expression		χ^2^	*P* value
	Low (N=499)	High (N=502)		
**Age (years)**				
<60	111	117	0.190	0.663
≥60	381	376		
**Gender**				
Female	250	149	43.525	<0.001^*^
Male	249	353		
**T stage**				
T1+T2	425	416	1.157	0.282
T3+T4	72	85		
**N stage**				
N0	340	303	8.784	0.003^*^
N1+N2+N3	146	194		
**M stage**				
M0	342	402	0.632	0.427
M1	17	15		
**TNM stage**				
I-II	398	394	0.406	0.524
III-IV	94	103		
**Status**				
Alive	353	316	6.857	0.009^*^
Dead	146	186		

*, *P*<0.05; NSCLC, non-small cell lung cancer; TNM stage, tumor-node-metastasis stage.

**Table 3:** Univariate and multivariate analysis of OS in patients with NSCLC.

Characteristics	Univariate analysis				Multivariate analysis			
	*P* value	HR	95%CI		*P* value	HR	95%CI	
Age (≥60/<60 years)	0.137	1.231	0.936-1.619					
Gender (Male/Female)	0.078	1.225	0.977-1.537					
TNM stage (III-IV/I-II)	<0.001^*^	2.081	1.639-2.642					
N stage (N1+N2+N3/N0)	<0.001^*^	1.708	1.372-2.125		0.007^*^	1.426	1.102-1.845	
T stage (T1+T2/T3+T4)	<0.001^*^	1.929	1.480-2.514		0.004^*^	1.581	1.155-2.166	
M stage (M1/M0)	0.004^*^	2.107	1.268-3.499					
KIAA0101 expression (High/Low)	0.044^*^	1.250	1.006-1.553		0.049^*^	1.249	1.001-1.559	

*, *P*<0.05; OS, overall survival; HR, hazard ratio; 95%CI, 95% confidence interval; TNM stage, tumor-node-metastasis stage

## Abbreviations

PCNA: proliferating cell nuclear antigen
NSCLC: non-small lung cancer
TCGA: The Cancer Genome Atlas
GEO: Gene Expression Omnibus
IHC: immunochemistry
qRT-PCR: quantitative real-time PCR
IF: immunofluorescence
OS: overall survival
HR: hazard ratio
95%CI: 95% confidence interval
ROC: receiver operating characteristic
AUC: area under the curve
HCC: hepatocellular carcinoma
cDNA: complementary DNA
FFPE: formalin-fixed paraffin-embedded
OD: optical density
GSEA: gene set enrichment analysis
NES: normalized enrichment score
FDR: false discovery rate
CTLA-4: cytotoxic T-lymphocyte-associated antigen 4
PD-1: programmed death 1
OEATC-1: overexpressed in anaplastic thyroid carcinoma-1
AFP: alpha-fetoprotein
CEA: carcinoembryonic antigen
EMT: epithelial-to-mesenchymal transition
TNM stage: tumor-node-metastasis stage
